# An open access dataset for developing automated detectors of Antarctic baleen whale sounds and performance evaluation of two commonly used detectors

**DOI:** 10.1038/s41598-020-78995-8

**Published:** 2021-01-12

**Authors:** Brian S. Miller, The IWC-SORP/SOOS Acoustic Trends Working Group, Brian S. Miller, Kathleen M. Stafford, Ilse Van Opzeeland, Danielle Harris, Flore Samaran, Ana Širović, Susannah Buchan, Ken Findlay, Naysa Balcazar, Sharon Nieukirk, Emmanuelle C. Leroy, Meghan Aulich, Fannie W. Shabangu, Robert P. Dziak, Won Sang Lee, Jong Kuk Hong

**Affiliations:** 1 Australian Antarctic Division, 203 Channel Highway, Kingston Tasmania, Australia; 2 Unaffiiliated, Brisbane, QLD Australia; 3 Pacific Marine Environmental Laboratory, NOAA, Newport, OR USA; 4 Unaffiliated, Bretagne, France; 5 Centre for Marine Science and Technology, Curtin University, Bentley, WA Australia; 6 Department of Environment, Forestry and Fisheries, Fisheries Management Branch, Cape Town, South Africa; 7 Mammal Research Institute Whale Unit, University of Pretoria, Hatfield, Private Bag X20, Pretoria, 0028 South Africa; 8 Korea Polar Research Institute, Incheon, 21990 South Korea; 9 Applied Physics Lab, University of Washington, Seattle, WA USA; 10 Alfred-Wegener Institute Helmholtz Centre for Polar and Marine Research, Bremerhaven, Germany; 11 Centre for Research into Ecological and Environmental Modelling, University of St Andrews, St Andrews, Scotland, UK; 12 ENSTA Bretagne Lab-STICC UMR CNRS 6285, Paris, France; 13 Department of Marine Biology, Texas A&M University at Galveston, Galveston, TX USA; 14 COPAS Sur-Austral Department of Oceanography, Universidad de Concepcion, Concepción, Chile; 15 Centre for Sustainable Oceans, Cape Peninsula University of Technology, Cape Town, South Africa

**Keywords:** Ecology, Ecology, Marine biology, Scientific data

## Abstract

Since 2001, hundreds of thousands of hours of underwater acoustic recordings have been made throughout the Southern Ocean south of 60° S. Detailed analysis of the occurrence of marine mammal sounds in these circumpolar recordings could provide novel insights into their ecology, but manual inspection of the entirety of all recordings would be prohibitively time consuming and expensive. Automated signal processing methods have now developed to the point that they can be applied to these data in a cost-effective manner. However training and evaluating the efficacy of these automated signal processing methods still requires a representative annotated library of sounds to identify the true presence and absence of different sound types. This work presents such a library of annotated recordings for the purpose of training and evaluating automated detectors of Antarctic blue and fin whale calls. Creation of the library has focused on the annotation of a representative sample of recordings to ensure that automated algorithms can be developed and tested across a broad range of instruments, locations, environmental conditions, and years. To demonstrate the utility of the library, we characterise the performance of two automated detection algorithms that have been commonly used to detect stereotyped calls of blue and fin whales. The availability of this library will facilitate development of improved detectors for the acoustic presence of Southern Ocean blue and fin whales. It can also be expanded upon to facilitate standardization of subsequent analysis of spatiotemporal trends in call-density of these circumpolar species.

## Introduction

Underwater passive acoustic monitoring (PAM) for marine mammals is a fast-growing field due to increased availability of, and flexibility in deploying, underwater recording devices^1–3^. PAM has especially high potential to provide information about marine mammals in remote or difficult to access areas, such as Antarctic waters. Since 2001, hundreds of thousands of hours of long-term acoustic recordings that span many years have been collected throughout the Southern Ocean. Many of these recordings were made for the purposes of learning about two endangered species that are especially detectable by PAM: Antarctic blue whales (*Balaenoptera musculus intermedia*) and fin whales (*B. physalus)*^4^.

### Monitoring blue and fin whales in the Southern Ocean

Historically, blue whales were heavily exploited throughout the Southern Ocean. Approximately 360,000 blue whales were caught across the Southern Hemisphere in the mid-twentieth century, depleting the population to less than 1% of their pre-whaling population^5^. The most recent abundance estimate of Antarctic blue whales suggest that the population contained between 1140 and 4440 individuals and may be slowly increasing at a rate between 1.6 and 14.8% per year (95% CI; mean of 8.2%), however this estimate was for the Antarctic summer of 1997/98, and thus is now dated by more than 20 years^6^. Over 725,000 fin whales were caught during the twentieth century^7^, yet circumpolar abundance of Southern Ocean fin whales has never been estimated since extant data sources are not sufficient to do so with fidelity^8^.

Given the endangered status of both blue and fin whales globally, the critically endangered status of Antarctic blue whales^9^, and the fact that both are long-lived species that are believed to reproduce every 2–3 years^10^, long-term monitoring is imperative to examine population trends and the effectiveness of current conservation measures (e.g. the moratorium on commercial whaling; https://iwc.int/commercial). PAM from fixed sensors is an ideal method for obtaining cost-effective broad spatial and long-term temporal coverage of blue and fin whale occurrences throughout a vast Southern Ocean region that is challenging to access. Blue and fin whales each produce distinct calls that can be repeated as songs, or produced as individual notes, which are unique to their respective species. In the case of Antarctic blue whales some sounds are unique to their population^11–13^. The repeated, loud, low-frequency, and long-travelling calls from these endangered species provide an extremely efficient means of identifying the presence of whales in the remote Antarctic waters of the Southern Ocean.

The Antarctic Blue and Fin Whale Acoustic Trends Project started in 2009 as one of the original projects of the International Whaling Commission’s Southern Ocean Research Partnership (IWC-SORP; https://iwc.int/sorp), and in 2017 expanded further to become a capability working group of the Southern Ocean Observing System (SOOS; www.soos.aq). The overarching goal of this project is to use acoustics to examine trends in Antarctic blue and fin whale population growth, abundance, distribution, seasonal movements and behaviour.

Through IWC-SORP, the Acoustic Trends Project working group has built upon the pioneering work done in the first decade of the twenty-first century, and has fostered an increasing number of passive acoustic studies focusing on the calls of Antarctic blue whales and to a lesser extent fin whales across broad time and spatial scales, as well as acoustic data processing and analysis methodology. The data from these studies have been collected both from ships during Antarctic voyages and from long-term moored recording devices^4,14–31^. The project working group presently (as of March 2020) has access to more than 300,000 h of passive acoustic data that have been collected throughout the Southern Ocean over the past 20 years.

### Automated detection of whale sounds

The volume of existing and incoming acoustic data far exceeds the capacity of human expert analysts to manually inspect it, and as a result automated algorithms have been relied upon to determine the presence of sounds from marine mammals in the recordings. Ecological results from long-term analyses have been reported in the form of presence (e.g. months, days, or hours of recordings with call presence), or as estimates of call numbers per time-period (e.g. see studies listed in Table 1). However, the results are not easily comparable because different studies had different data collection protocols and employed different analytical techniques, neither of which have been standardised. Furthermore, robust measures of bias and variability, which can be dataset-specific, are not always reported alongside results (Table 1).

**Table 1:** Previous analyses of long-term datasets that have used automated algorithms to detect calls and report spatial distribution and/or temporal occupancy of Antarctic blue whales.

Study	Detection method (software used)	Noise pre-processing	True positive rate	False positive rate	False positive removal	Characterisation/validation summary
Širović et al. 2004^17^	Spectrogram correlation (Ishmael) Energy sum (Ishmael)	Not reported	Not reported	< 1%	No	Threshold was iteratively adjusted until false positive rate was < 1%. Calls on days with fewer than 50 detections were inspected
Širović et al. 2009^18^	Spectrogram correlation (Ishmael)	Not reported	Not reported	Not reported	All	Visual inspection of all detections to remove false positives
Samaran et al. 2013^30^	Spectrogram correlation (XBAT)	Not reported	Not reported	6%^a^	Some	Months with fewer than 50 detections: visual inspection of detections to remove all false positives. Otherwise 10% of randomly selected detections inspected
Tripovich et al. 2015^20^	Energy detection (Ishmael)	Not reported	Not reported	14.6%	All	Visual inspection of all detections to remove false positives
Thomisch et al*.* 2016^19^	Spectrogram correlation (custom developed)	Not reported	Not reported	Nominally < 1%	No	False detection rates and thresholds determined via detection function quantiles from 100 randomly selected detections^b^
Leroy et al*.* 2016^26^	Subspace projection detection^36^	Noise-adaptive threshold	Not reported^c^	Nominally < 3%	Some	Detections deemed false if the frequency at maximum amplitude was different than that of unit-A
Balcazar et al*.* 2017^45^	Energy sum (Ishmael)	Not reported	93.3–97.3%	14.6–98.9%	All	Comparison against expert human observer who annotated 1 randomly selected day each month for each site. Visual inspection of all detections to remove false positives
Buchan et al*.* 2017^42^	Spectrogram correlation (Ishmael)	Not reported	99.998%^d^	Not reported	All	20% subset of days with no automated detections visually inspected to determine false negative rate. Visual inspection of all detections to remove false positives
Shabangu et al. 2017^16^	Spectrogram correlation (XBAT)	Not reported	42–83%	Not reported	All	Visual inspection of entire dataset (1518 h) to assess remove false positives and include missed detections

^a^False positive rate reported only for months when there were more than 500 calls detected.

^b^False positive detections were from a different detector operating in an adjacent frequency band with a similar, frequency-adjusted, spectrogram correlation kernel.

^c^True positive rates for this detector for high, medium, and low signal to noise ratio (SNR) calls and a variety interfering noises reported by Socheleau et al. 2015, but the prevalence of these conditions within the full dataset is not indicated.

^d^False negative rate reported as a percentage of total uncorrected detections for a 20% subset of days without automated detections.

A variety of automatic detection algorithms have been used to detect the calls of blue whales and fin whales. Algorithms to detect stereotyped calls of these species include matched filters^32–34^, energy detectors^35^, subspace projection detectors (blue whales only^26,36^). However, the most widely used algorithm has been spectrogram correlation^37^, and this has been implemented in a variety of software packages^38–40^ and has been used widely on a variety of different datasets^16–20,30,41–44^. Spectrogram correlation is similar to matched filtering except that it acts on the spectrogram, rather than purely in the time or frequency domains; instead of cross-correlating a time series or spectrum, it correlates an image template or kernel pixel-by-pixel with the spectrographic data of interest.

### Factors that affect the detector performance

Three main factors can impact the performance of an automated detection algorithm: acoustic properties of the recording site, variability in signals that are being detected, and variability in the characteristics of the recording system. The acoustic recordings from the Southern Ocean span a wide geographic and temporal range and encompass a variety of environments, thus characteristics of the recording site (e.g. propagation loss and noise levels) are expected to be both site- and time-specific^43,46^.

In addition to site-specific features, the properties of blue and fin whale sounds can change over time and space. Sounds from most blue whale populations have changed slowly and in a predictable manner since they were first described in the 1970s^14,47–49^. On top of the well documented decreases in tonal frequency of sounds from year-to-year, there are predictable intra-annual changes that have also been observed^14,48,50^. There is some evidence that the properties of fin whale sounds vary geographically in the Antarctic^15,18^, and have been found to vary temporally in other oceans^33,51,52^. While these changes may seem small and/or occur over long time periods, they must nevertheless be accounted for when using automated detection algorithms to detect trends in long-term and widely dispersed datasets^53^.

Lastly, the acoustic recordings around the Antarctic have been made with a variety of instruments. These include: Scripps *Acoustic Recording Packages* (ARP); Multi-Electronique *Autonomous Underwater Recorder for Acoustic Listening (*AURAL); Australian Antarctic Division *Moored Acoustic Recorders* (AAD-MAR), Develogic *Sono.Vaults*; and Pacific Marine Environmental Laboratory—*Autonomous Underwater Hydrophones* (PMEL-AUH). Different instruments may have different capabilities, including depth rating, system frequency response, and duty cycle requirements, and these further affect the performance of an automated detector^54^. For example, the duty cycle of an instrument, for example, is known to affect the accuracy of predicting the presence of Antarctic blue whales in addition to the call rate^55^. Additionally, the depth of the recorder is expected to change the detection range and noise levels observed at a recorder^4^.

Here we create and document an open access set of recordings collected around the Antarctic and manual annotations of blue and fin whale call occurrences in a subset of those recordings. This dataset takes the form of an “annotated library” of Antarctic underwater sound recordings. We demonstrate how the library can be used to evaluate the performance of automated detectors over the variety of recording scenarios contained within the library. We also suggest methods to help standardise the reporting of results with a view towards facilitating long-term comparisons of PAM studies of baleen whales around Antarctica.

## Methods

### Towards a representative circumpolar dataset

**Figure 1:**
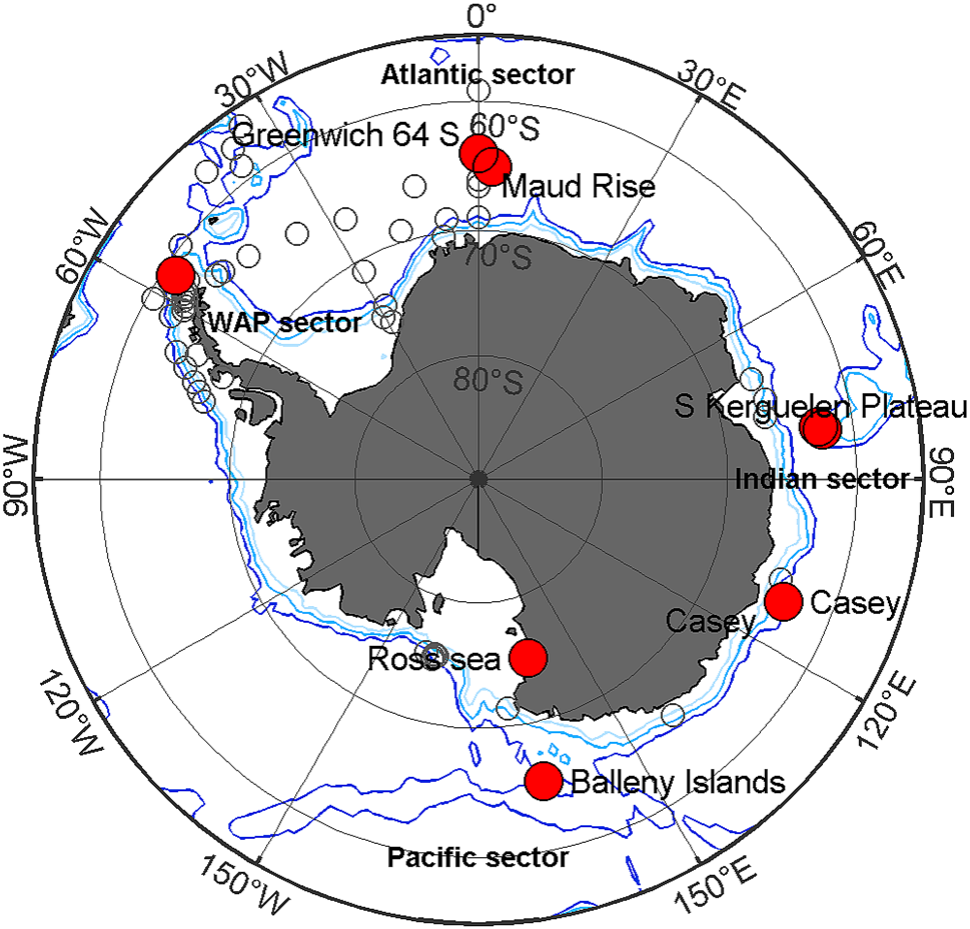
Map of Antarctic underwater recording sites illustrating sites used in this study (red circles) and known locations of long-term recordings from 2001 to 2017 (open circles). Map created using M_Map version 1.4k^56^ and ETOPO 1 bathymetry (https://www.eoas.ubc.ca/~rich/map.html). Light, medium, and dark blue lines show 1000, 2000, and 3000 m depth contours respectively.

Our annotated library contains data from four geographic regions: the Atlantic, Pacific, and Indian sectors of the Southern Ocean and the Western Antarctic Peninsula (WAP; Fig. 1). In each region, we identified sites that had at least a full year of data from 2014 or 2015, and ideally had two consecutive years. When two consecutive years were not available, another year from the same site was included or two different sites were selected. The Indian sector site also included data from 2005 to increase the temporal span of the library, as well as a second location with data from 2014 and 2017. The data in this library were recorded using a variety of instruments: ARP, AURAL, AAD-MAR, Sono.Vault; and PMEL-AUH (Table 2).

**Table 2:** Description of annotated datasets including the site-year, location, initials of the analyst who made the annotations, type of instrument, start and stop date of the annotations, and number of hours (independent dates and times) annotated, as well as the total duration of the recordings annotated.

Dataset name (site-year)	Latitude	Longitude	Analyst	Instrument	Start	Stop	Unique calendar hours with annotations	Unique calendar days with annotations	Audio duration annotated (h)
Maud Rise 2014	65° 0.00′ S	2° 30.00′ E	BSM	AURAL	2014-01-12	2014-09-17	201	201	83.3
Greenwich 64S 2015	64° 00.32′ S	0° 00.22′ E	NB	Sono.Vault	2015-01-02	2015-12-31	190	190	31.7
S Kerguelen Plateau 2005	62° 35.44′ S	81° 15.64′E	NB	ARP	2005-01-31	2006-01-31	200	200	200
S Kerguelen Plateau 2014	62° 22.81′ S	81° 47.81′ E	NB	AAD-MAR	2014-02-22	2015-02-20	200	200	200
S Kerguelen Plateau 2015	“	“	NB	AAD-MAR	2015-02-10	2016-01-27	200	200	200
Casey 2014	63° 47.73′ S	111° 47.23′ E	NB	AAD-MAR	2013-12-25	2014-12-12	194	194	194
Casey 2017	“	“	MA	AAD-MAR	2016-12-15	2017-11-07	185	185	185
Ross Sea 2014	75° 01.19′ S	164° 35.51′ E	SN	PMEL-AUH	2014-02-08	2014-12-14	176	176	176
Balleny Islands 2015	65° 21.34′ S	167° 54.69′ E	SN	PMEL-AUH	2015-01-15	2016-01-10	205	205	204
Elephant Island 2013	61° 00.88′ S	55° 58.53′ W	NB	AURAL	2013-01-12	2013-12-02	2247^a^	93^a^	187^a^
Elephant Island 2014	“	“	ECL	AURAL	2014-01-01	2014-12-31	2592	108	216

^a^Not evenly distributed throughout the year.

### Subsampling from each dataset

Moorings in the Antarctic are typically recovered and serviced at the most once a year due to their remote locations, potentially long periods of ice cover, and reduced/negligible access during Antarctic winter. Thus we define a site-year as a recording from a single instrument and site that is approximately a year in duration. A subset of approximately 200 h of data was selected from each site-year for annotation. This number of hours was chosen a priori and was constrained by budgetary limits, but it was believed to be a reasonable trade-off among analyst time, maintaining adequate sample sizes within each site-year, and annotating a sufficient number of different site-years.

For each site-year a systematic random subsampling scheme was used to generate a representative set of acoustic recordings from the larger dataset. The systematic random subsampling scheme consisted of:

1. splitting the dataset into “chunks” of time. The optimal length of a time chunk will be species and study specific. For the annotated library time was split into mostly hour-long chunks with some exceptions.
2. calculating the spacing between chunks, *t*_*s*_ to ensure that the desired sample size of time chunks is created and that there was broad representation of hours in the day across all chunks. For the annotated library, spacing was calculated such that there were at least 150, and usually nearer to 200, annotated periods between the first and last available chunks.
3. Picking a random number between 1 and *t*_*s*_, the spacing, to determine the starting chunk (this was the random element of the subsampling scheme).

The aim of the subsampling scheme was to capture a representative sample of the signals recorded for a given site-year, i.e., to select periods of time with calls that spanned a range of signal-to-noise ratios (SNR) and periods of time without calls, as well as other sounds that might contribute to false positives (though rare events may have been missed). Having a temporally representative subsample of sounds was deemed necessary to understand how a detector would perform when used across the entire dataset.

**Figure 2:**
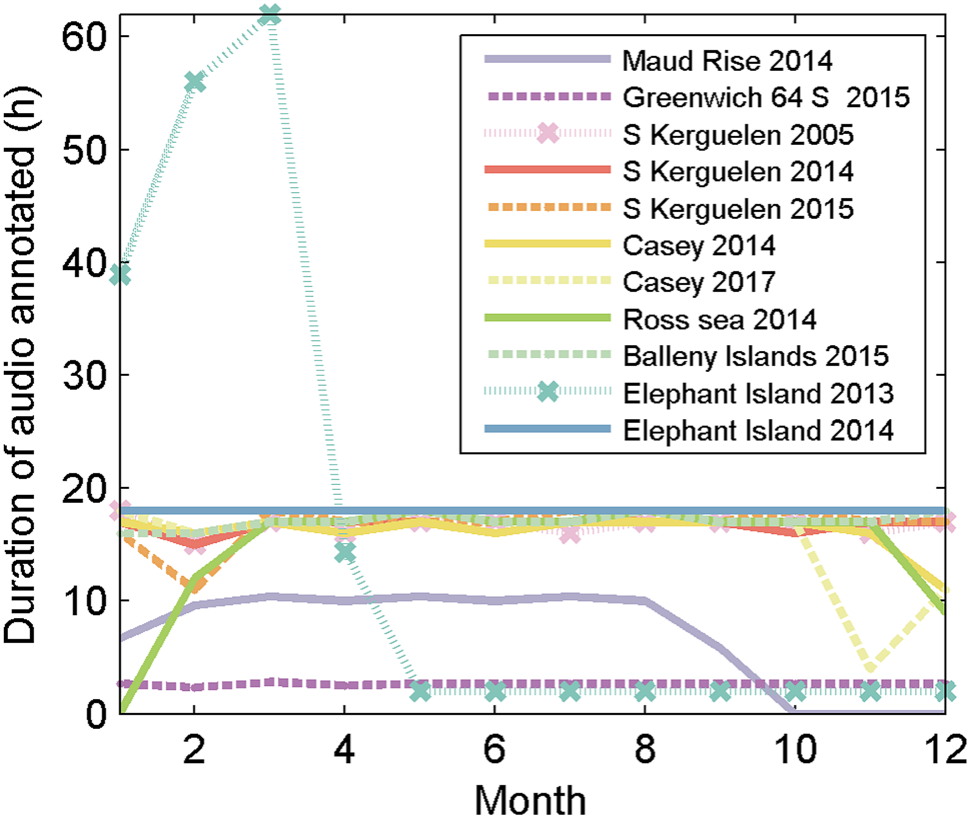
Number of hours annotated for each month for each site-year.

For each site, 10–18 h of data were annotated per month with the exceptions of the Ross Sea in 2014 which had no data for January, and Maud Rise 2014 which recorded only from Jan-Sep (Fig. 2). Over the whole year the subsampling scheme ensured a relatively even distribution of hours across a 24 h cycle, with each site containing between 5 and 10 h inspected for any given hour in the cycle.

However, four sites had been annotated previously, and thus used slightly different subsampling schemes. Additionally, three of these sites had recording duty cycles shorter than an hour. Elephant Island 2013 and 2014 had a duty cycle of 5 min/h. Maud Rise 2014 had a duty cycle of 25 min/hour. For Elephant Island 2014 9 days per month were selected (randomly, but with roughly even spacing between them), and all 5 min segments were analysed for selected days (total duration of all audio segments 216 h). For Maud Rise 2014 200 independent evenly spaced hours were selected and each 25 min segment was analysed (total duration of all audio segments 83.3 h). For Elephant Island 2013 all 5 min segments for every day were analysed from 12 Jan 2013 to 8 Apr 2013. For the remaining months (May–Dec 2013) all segments from one day each month were analysed. Lastly, Greenwich 64 S 2014 had 10 min long sub-samples that were annotated. These were spread over 190 unique hours throughout the year (31.6 h audio duration).

### Manual annotations

For manual detection and annotation of calls, recordings were visualised in Raven Pro 1.5^57^. Spectrogram details included a 120 s timespan, frequency limits between 0 and 125 Hz, Fast Fourier Transform (FFT) of approximately 1 s in duration; frequency resolution of approximately 1.4 Hz, and 85% time overlap between successive FFTs. Lower and upper limits of the spectrogram power (spectrogram floor and ceiling) were adjusted for each 1-h segment. The lower limit of spectrogram power was adjusted by the analyst until approximately 25% of the spectrogram was at or below the floor value (i.e. a visual estimate of 25th percentile spectral noise level). The ceiling of the spectrogram was then adjusted so that the difference between ceiling and floor was between 30 and 50 dB relative to full-scale. The ceiling of the spectrogram could then be adjusted further to provide additional contrast in the event of long loud broadband sounds such as ice or prolonged occurrence of baleen whale choruses.

**Figure 3:**
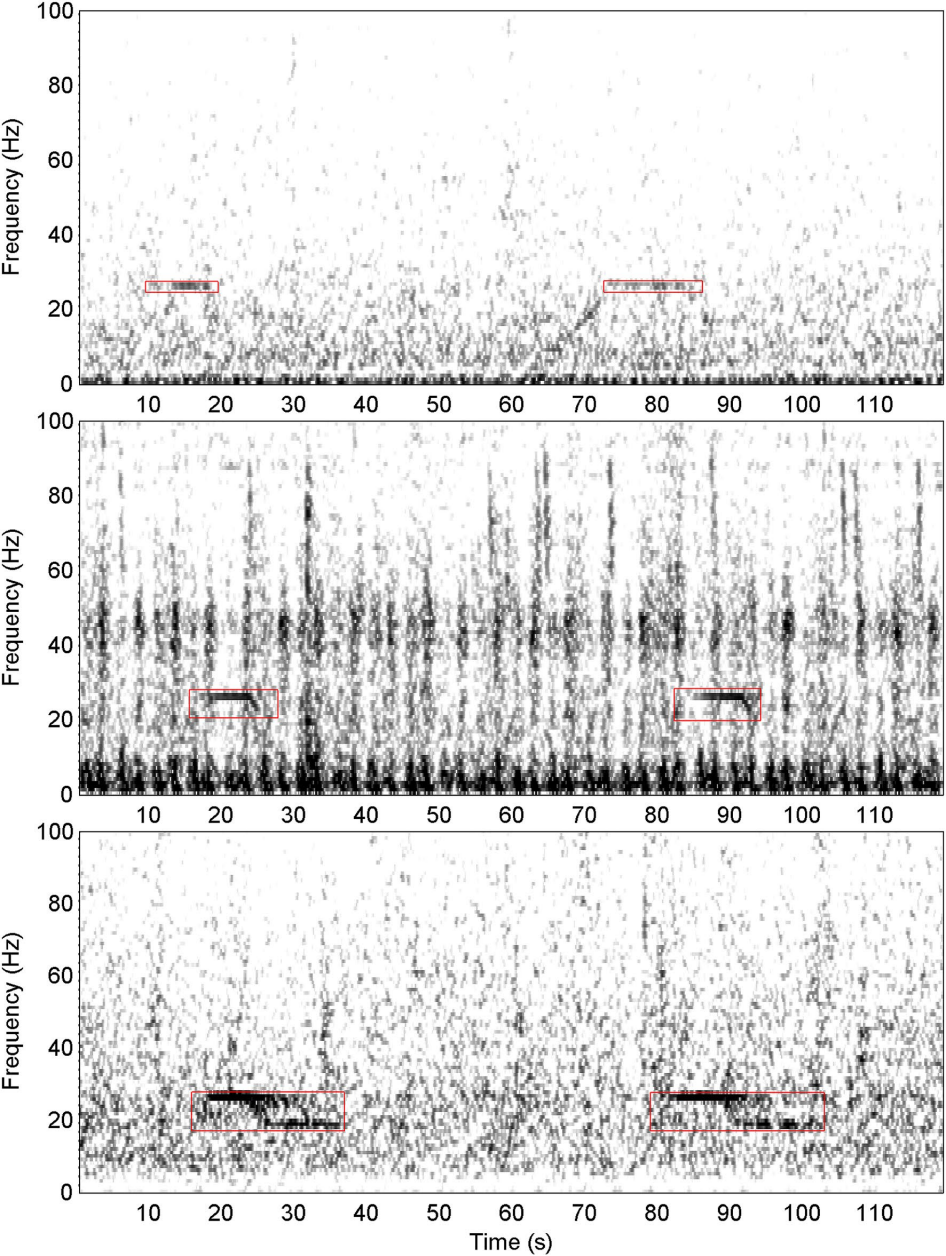
Spectrograms showing examples of Bm-Ant-A (top); Bm-Ant-B (middle); and Bm-Ant-Z (bottom). All spectrograms used a sample rate of 250 Hz, 256 point FFT with 85% overlap. Bm-Ant-A example is from site-year Balleny Islands 2015 and starts at 25-Feb 07:24:52. Bm-Ant-B example is from site-year Elephant Island 2014 starting at 20-Jan 19:00:45. Bm-Ant-Z example is from site-year S Kerguelen Plateau 2014 and starts at 01-Mar 12:25:10. Red boxes are indicative of time–frequency boundaries of manual annotations.

**Figure 4:**
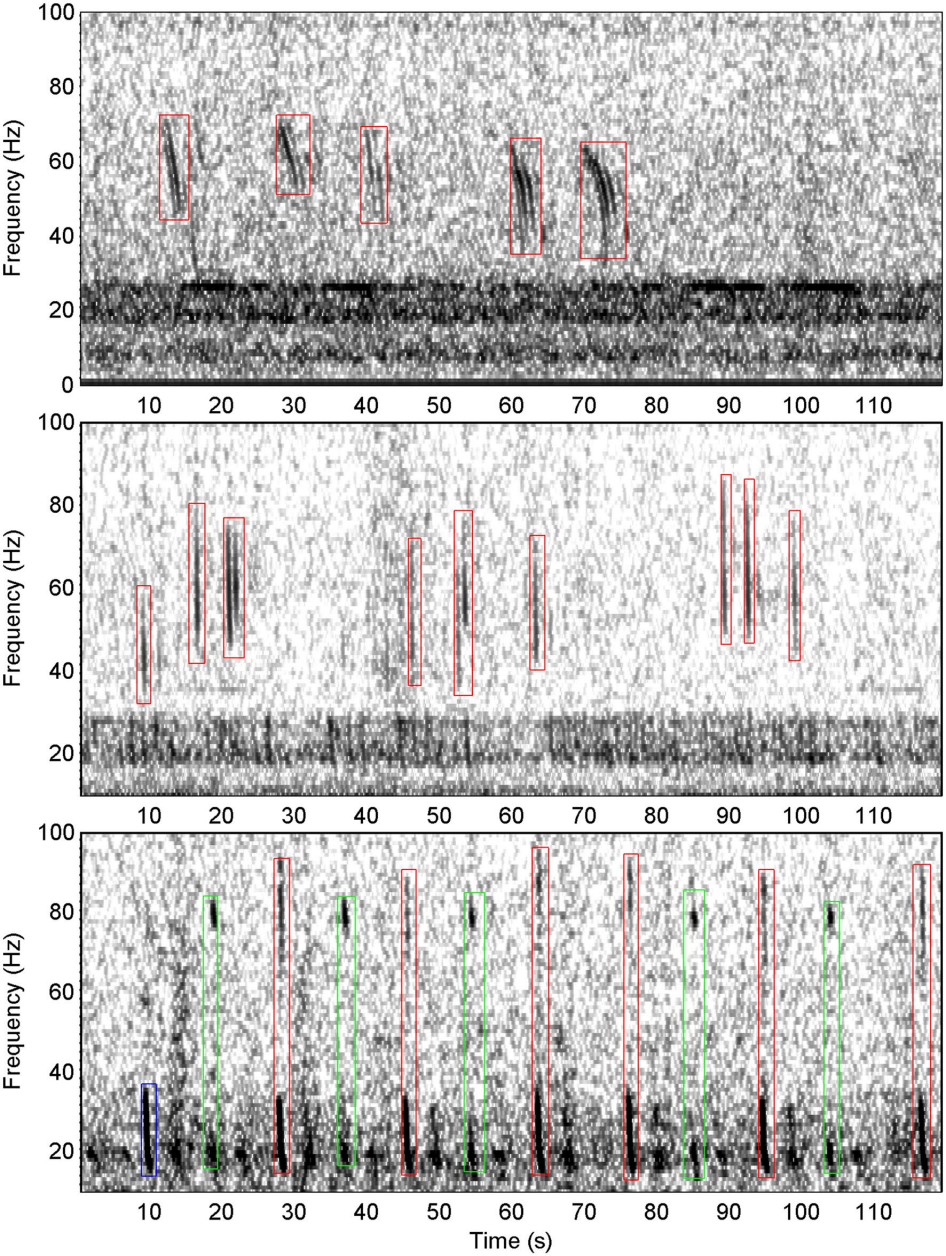
Spectrograms showing Bm-D (top); Bp-Downsweep (middle) and Bp-20 Hz (bottom panel blue box) along with two forms of Bp-20Plus (bottom panel red and green boxes). All spectrograms in this figure used a sample rate of 250 Hz, 256 point FFT with 85% overlap. A chorus of blue and fin sounds is visible in the top and middle panels from 20–30 Hz. Bm-D spectrogram is from S Kerguelen Plateau and starts at 2015-04-16 19:18:00. Bp-Downsweep spectrogram is from S Kerguelen and starts at 2005-04-24 00:05:00. Bp-20 Hz and Bp-20Plus spectrogram is from Balleny Islands and starts at 2015-03-22 00:50:10.

Within each subsample the analyst marked the time–frequency bounds of all occurrences of blue and fin whale sounds. Each analyst had extensive expertise in the identification of blue and fin whale sounds, particularly those from the Southern Hemisphere including the Antarctic. The analyst assigned one of eight different classifications to annotations: Bm-Ant-A, Bm-Ant-B, Bm-Ant-Z, Bm-D, Bp-20, Bp-20Plus, Bp-Downsweep, and Unidentified. Detailed descriptions of each of these classifications (including citations) are provided in Table 3, Figs. 3, and 4. The first two letters of the classification correspond to genus and species, so sounds starting with *Bm* were produced by blue whales and *Bp* by fin whales. The remainder of the classification corresponds to particular call types for that species (or sub-species in the case of Antarctic blue whales).

**Table 3:** Classification and labelling system for blue and fin whale sounds in the SORP library of annotated recordings.

Label	Call Type	References	Description
Bm-Ant-A	Antarctic blue whale unit A	^11,27^	A constant frequency tone between 28 and 25 Hz (depending on the year) without other units
Bm-Ant-B	Antarctic blue whale unit AB	^11,27^	Antarctic blue whale unit A tone followed by partial or full inter-tone downsweep (unit B)
Bm-Ant-Z	Antarctic blue whale z-call; (AKA 3 unit vocalisation)	^11,17^	Antarctic blue whale 'z-call' with upper tonal unit A and lower tonal unit C present (and downswept unit B either present or absent)
Bm-D	Blue whale FM (AKA D-calls)	^11^	Any downswept frequency modulated calls from blue whales. Typically, but not always, longer in duration and lower in frequency than FM calls from fin and minke whales
Bp-20 Hz	Fin whale 20 Hz pulse	^12^	20 Hz fin whale pulse without substantial energy at higher frequencies
Bp-20Plus	Fin whale 20 Hz pulse with energy at higher frequencies (e.g. 89 or 99 Hz components)	^15,18,24^	Fin whale 20 Hz pulse including secondary energy at higher frequencies (e.g. upper frequency peak near 80–100 Hz)
Bp-Downsweep	Fin whale FM calls (AKA ‘high frequency’ downsweep; AKA 40 Hz pulse)	^24,58,59^	Frequency modulated, usually downswept calls believed to be produced by fin whales. Usually, but not always shorter in duration and slightly higher in frequency than FM calls produced by blue whales
Unidentified	Unidentifiable sounds	Not applicable	Any transient biological sound that could not be confidently identified as any of the above classifications. This included sounds that could potentially be biological, but were substantially different than the above classifications. It also included FM downsweeps where the analyst was not certain whether they were blue whale D calls or fin whale downsweeps

In addition to marking the time–frequency boundaries of all potential detections, the analyst also noted qualitative information about background noise and other sources of sound that were present in each chunk that was inspected, including the presence and intensity of a “chorus” of elevated background noise in the 20–30 Hz band over which Antarctic blue whale z calls and fin whale 20 Hz pulses contain most of their energy^18,19^.

**Figure 5:**
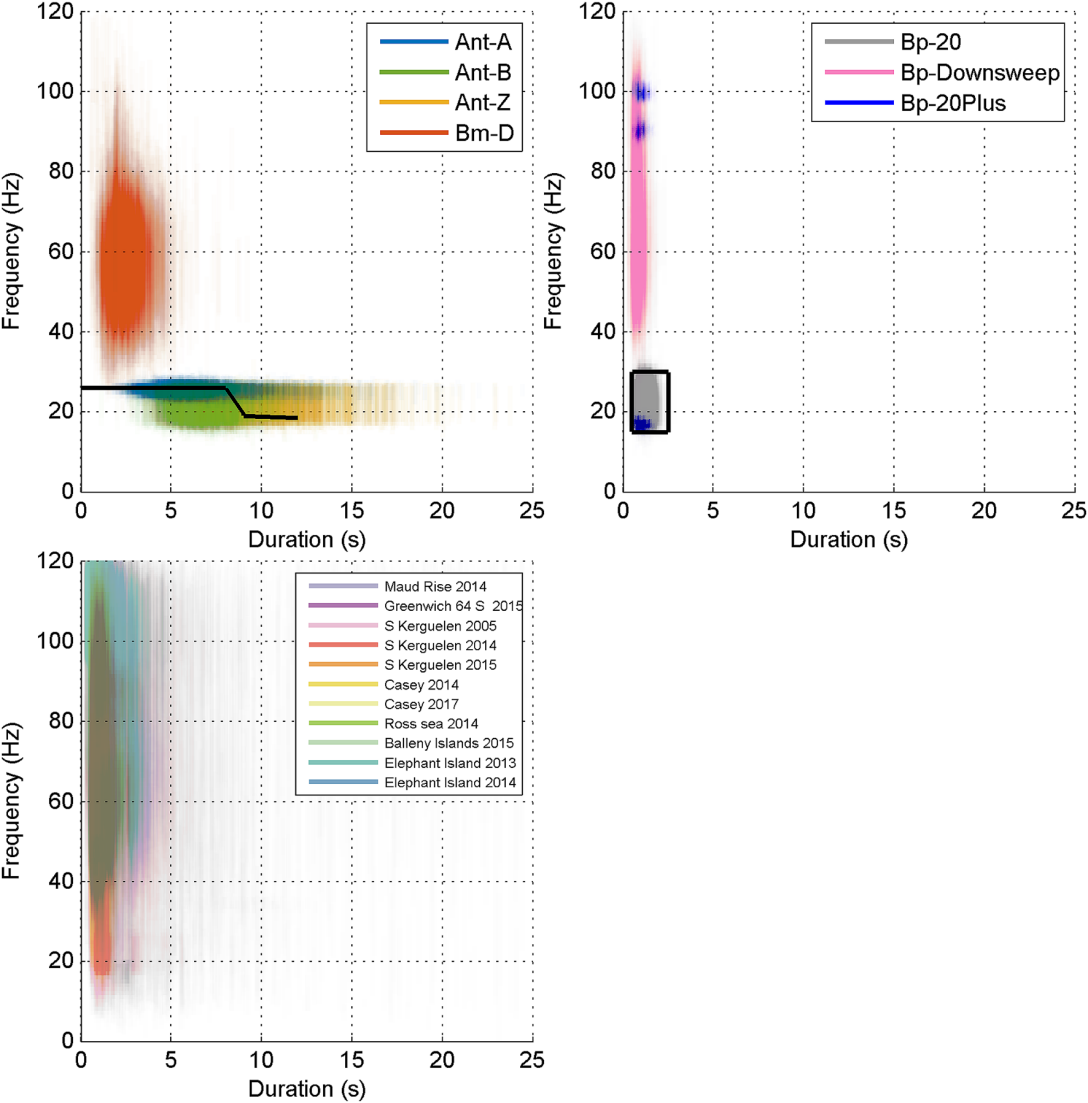
Duration (t_90%_) and frequency bounds (f_5%_ and f_95%_) of a subset of individual annotations plotted as 99.9% transparent lines. Top left: blue whale sounds for all site-years. Top right: fin whale sounds for all site-years, excluding ElephantIsland2014 (see “Discussion” for explanation of this exclusion). Black line and black box show the kernel of the spectrogram correlation detector and time–frequency bounds for the spectrogram energy-sum detector respectively. For clarity, fin whale 20 Hz with higher frequency components (Bp-20Plus) are shown as point clouds at the minimum and maximum frequencies rather than vertical lines. Bottom left: unidentified sounds with colours representing each site.

For each site and classification, the 5th and 95th percentile frequency limits and durations of annotations were measured and plotted to visually identify gross differences among sites as a rough form of “quality control” across sites and analysts. These percentiles also directly informed respective parameters for automated detectors (Fig. 5).

Signal-to-noise ratio, as described by Lurton (2010)^60^, was then measured for each manual annotation. In brief, the root mean square (RMS) signal and noise power, Z_s+n_, was measured for the full duration of each detection over the frequency band of interest: 17–29 Hz for Bm-Ant-A, Bm-Ant-B, Bm-Ant-Z; 20–30 Hz for Bp-20 Hz, Bp-20Plus. Since some analysts marked time–frequency boundaries more tightly than others, a buffer of 1 s before and after the observation was then created to ensure that no residual signal was included in the measurement of noise. The noise measurement period was the same duration as the annotation, but split evenly before and after the buffer (i.e. the noise period was *d*/2 s before and *d*/2 s after the buffer, where *d* is the duration of the manual annotation). RMS noise power, Z_n_ and variance of noise power, $\sqrt {{\Sigma }_{n}^{2} }$ was measured for t_noise_ over the same band of interest. Finally, the SNR in dB was calculated as:

1 $$SNR = 20\log_{10} \frac{{\left( {Z_{s + n} - Z_{n} } \right)^{2} }}{{\sqrt {{\Sigma }_{n}^{2} } }}$$

### Automated detectors

In order to demonstrate the utility of the annotated library and compare the site-specific performance of automated detection algorithms, we characterised the performance of two automated detectors commonly used for detecting sounds of Antarctic blue and fin whales for each of the sites in the annotated library: an energy sum detector, and a spectrogram correlation detector. Energy sum detectors rely only on knowledge of the duration and frequency band of the call, so in general can be more flexible if calls are variable within the band of detection. The spectrogram correlation detectors relies on a priori knowledge of the shape of the call in the time–frequency domain, and thus perform better when calls are highly stereotyped with relatively little variation in shape from one call to the next. These two types of detectors were chosen because to demonstrate that the library was suitable for different types of detectors, and not because we believed they were optimal for their respective tasks.

For fin whale 20 Hz pulses (both Bp-20 Hz and Bp-20Plus classifications) we applied an energy sum detector^38^ which targeted the 20 Hz pulse of fin whales by summing the energy for each spectrogram slice in the band from 15 to 30 Hz. Thus, the detection score was the sum of the squared value of all spectrogram frequency bins (after noise normalisation) at that time step. In addition to the threshold for summed energy, a minimum and maximum time over threshold of 0.5 and 2.5 s as well as a minimum time between detections of 0.5 s were used as criteria for detection of individual fin whale 20 Hz pulses.

For Antarctic blue whale song (i.e. classifications of Bm-Ant-A, Bm-Ant-B, and Bm-Ant-Z), we applied a spectrogram correlation detector^37^ which targeted Bm-Ant-Z calls, but was also effective at detecting Bm-Ant-A and Bm-Ant-B since these call types are essentially each a subset of the full Z-call. The detection score for the spectrogram cross-correlation detector was the magnitude of the 2D cross-correlation between the correlation kernel and the spectrogram at each time step. Thus the values for threshold are in a somewhat arbitrary units of ‘recognition score’ which is the result of cross-correlation between normalised spectrogram and correlation kernel. In addition to a detection score threshold, a minimum time over threshold and minimum time between calls were also used^37^.

Detectors were run on the annotated library’s subsets of recordings for each site using Pamguard Version 2.01.03^39^. Each detector was applied to the subsample of data for each site using a range of thresholds (determined empirically) in order to create a receiver-operator characteristic (ROC) and precision-recall (PR) curve for each site^61,62^.

#### Feature extraction and detector design

The spectrogram correlation and energy sum detectors were parameterised by the time and frequency properties of calls, namely the duration and frequency of each unit of each call. The specific time–frequency properties that we used for each detector were chosen based on published descriptions of calls. The detector parameters were validated by simple comparison with measurements from manual annotations, specifically the 5th and 95th percentiles of the energy distribution for each annotation (Fig. 5).

The mean duration of all manual annotations for that classification, $\overline{ d}$, was used to determine the time boundaries for each automated detection. A “refractory period” of length $\overline{ d}$ was applied after each detection to prevent new detections from overlapping existing detections. The refractory period prevented multipath arrivals (e.g. reverberation from the seabed and surface that can arrive before or after the detection) from being detected by the automated detector. However, the refractory period had the downside of preventing legitimate detection of calls from different animals that arrived within $\overline{ d}$ seconds of each-other. This was believed to be a prudent trade-off because multipath arrivals appeared to be far more common than overlapping calls from two different animals. Furthermore, by preventing automated detections from overlapping, the total number of possible automated detections (and true negative/false positive rates) could be calculated from the total duration of the recording,$\overline{ d}$, refractory period, and total duration of all the manual annotations.

Noise normalisation was applied to the spectrogram prior to automated detection. The noise normalisation algorithm was Pamguard’s ‘Average Subtraction’ algorithm, and this involved subtracting a decaying average for each spectrogram frequency bin at each time step. Specific parameters for the fin whale 20 Hz pulse detector are described in Table 4 and Antarctic blue whale song in Table 5.

**Table 4:** Parameters for the energy detector for Bp-20 Hz, Plus.

Lower frequency bound (Hz)	15
Upper frequency bound (Hz)	30
Thresholds	0.5, 1, 2, 3, 4, 5, 8, 16, 32, 64
Minimum time over threshold (s)	0.5
Maximum time over threshold (s)	2.5
Minimum time before next detection (s)	1
FFT time resolution (s)	1.024
FFT frequency resolution (Hz)	0.98
Noise normalisation type	Average subtraction
Average subtraction: Update constant	0.01

**Table 5:** Parameters for the spectrogram correlation detector for Bm-Ant-A,B,Z.

Unit A duration (s)	8.0
Unit B duration (s)	2
Unit C duration (s)	2
Unit A frequency (Hz)	*f*_*a*_ (see Eq. 2)
Unit B high frequency	*f*_*a*_ (see Eq. 2)
Unit B low frequency (Hz)	19.5
Unit C high frequency (Hz)	19.0
Unit C low frequency (Hz)	18.5
Thresholds	20, 40, 80, 160, 320, 640, 1280, 2560, 5120, 10,240
Minimum time over threshold (s)	3
Minimum time before next detection (s)	13
FFT time resolution (s)	1.024
FFT frequency resolution (Hz)	0.98
Noise normalisation type	Average subtraction
Average subtraction: update constant	0.001

To parameterise the blue whale detector the equation

2 $$f_{a} = 27.6659 - \left( {\frac{0.135}{{365}}} \right)t$$

was used to determine the frequency (in Hz) of unit A of Antarctic blue whale calls. In this equation, derived from^14^, *f*_*a*_ is the frequency of unit A, and *t* is the number of days since 12 March 2002. For each site-year *t* was set to be the 1^st^ of June for detector parameters that required estimation of *f*_*a*_.

#### Evaluation of detector performance

Detections from the automated detectors were matched to the human analyst by comparing the start and end times of all pairs of manual and automated detections. Detections were considered a match if there was any time overlap between manual and automated observations. This criterion created the potential for duplicate matches between multiple automated and manual annotations. Duplicates were identified and labelled, but were neither counted as true positives nor false positives when calculating ROC and precision-recall curves.

For each threshold automated detections were tabulated to create a confusion matrix of true positives, false positives, their respective rates, precision, and recall. ROC curves and precision recall curves for each site and detector were then created from each set of true and false positives (Fig. 7).

To investigate the relationship between the number of automated detections and SNR, a generalised additive model (GAM)^63^ was fitted using results from the automated detection process. For each manually detected call, SNR and whether or not the call was automatically detected was recorded. Specifically, each manual annotation was assigned a value of 1 when any automated detections matched, and a value of 0 when no automated detections matched. The matches were modelled as the response of logistic regression with SNR as a predictor using a GAM with a binomial family error distribution, a logit link function. The GAM was fitted separately for each site using the default number of knots within the package ‘mgcv’^63^ in R version 3.6.1^64^.

## Results

### Distribution of annotations throughout the library

The annotated library consisted of 1880.25 h (audio duration) of annotated data across 11 site-years and 7 sites. In total, there were 105,161 annotations across all sites, though the numbers of annotations were neither evenly distributed by site nor classification (Table 6). Bm-Ant-A was the most numerous annotation with 24,363 manual detections in total, while Bm-Ant-Z was the least numerous annotation with 2,515 manual detections in total. Ross Sea 2014 had the fewest annotations over all site-years with only 359 annotations (104 of Bm-Ant-A, and the remainder unidentified). Elephant Island 2014 had the most annotations of all site-years with 21,438 in total including unidentified sounds.

**Table 6:** Distribution and number of annotations at each site by classification type. Each cell in the table contains the percentage of hours (with the total number of manual annotations in brackets).

Site-year	Ant-A	Ant-B	Ant-Z	Bm-D	Bp-20	Bp-20 +	BpDownsweep	Unidentified
Maud Rise 2014	90.5 (2188)	10.5 (37)	9.5 (28)	5.5 (70)	3.0 (23)	1.0 (5)	2.0 (6)	42.0 (465)
Greenwhich 64 S 2015	80.5 (827)	47.4 (157)	12.6 (29)	10.5 (66)	1.1 (2)	0.5 (1)	4.7 (46)	57.9 (325)
S Kerguelen 2005	59.0 (812)	43.0 (237)	25.0 (166)	25.5 (435)	7.5 (788)	3.0 (78)	17.0 (444)	97.5 (3061)
S Kerguelen 2014	71.0 (2557)	60.5 (1177)	44.0 (563)	33.0 (435)	11.5 (1920)	11.0 (1826)	5.0 (344)	88.0 (4961)
S Kerguelen 2015	60.5 (1970)	49.5 (542)	24.5 (236)	28.5 (1180)	9.0 (552)	6.0 (718)	5.5 (344)	74.0 (1244)
Casey 2014	91.2 (3681)	77.3 (1398)	65.5 (1091)	41.2 (679)	2.1 (17)	0.0 (0)	0.0 (0)	96.9 (5648)
Casey 2017	69.5 (1741)	44.4 (558)	8.6 (119)	38.5 (553)	1.1 (78)	1.1 (214)	0.0 (0)	34.8 (130)
Ross Sea 2014	0.6 (104)	0.0 (0)	0.0 (0)	0.0 (0)	0.0 (0)	0.0 (0)	0.0 (0)	8.5 (255)
Balleny Islands 2015	30.2 (923)	5.9 (44)	2.9 (31)	3.4 (47)	9.3 (951)	1.0 (148)	3.9 (78)	1.5 (18)
Elephant Island 2013	37.4 (2625)	32.0 (1786)	4.7 (152)	7.2 (299)	16.0 (3662)	9.0 (1859)	4.8 (1042)	75.6 (22,927)
Elephant Island 2014	60.5 (6935)	15.5 (967)	2.5 (100)	10.0 (1034)	18.0 (4940)	11.3 (2912)	22.3 (3660)	10.6 (890)
Total	24,363	6903	2515	4798	12,933	7761	5964	39,924

The percentage of hours with each type of annotation was also variable across sites (Table 6). Bm-Ant-A had the highest percentage across all sites ranging from 0.6 to 91.2% of hours, while Bp-20Plus had the lowest proportions across all sites with no Bp20Plus detections at Casey 2014 or Ross Sea 2014. Antarctic blue whale classifications were generally present in higher percentage of hours than fin whale sounds across most site-years (Table 6).

### Description of classification features

Within each classification the 5th and 95th percentiles of the frequency bounds and durations were similar across sites, but with a few notable exceptions. Annotations of Bp-20 Hz, Bp-20Plus, and Bp-Downsweep from Elephant Island 2014, appeared to have longer durations than these classifications from other sites. However, visual comparison of these annotations suggest that this difference appeared to arise from the way the analyst marked annotations (i.e. more generous time-boundaries than other analysts) rather than true difference in the duration of the sound. This suggests that our use of the 90th percentile energy duration did not provide a measure of duration that was fully robust against analyst variability. Thus, different features or measures of duration may be more robust or appropriate for developing automated detectors and/or classifiers.

The stereotyped calls of Antarctic blue whales (Bm-Ant-A, Bm-Ant-B, and Bm-Ant-Z) and those of fin whales (Bp20Hz, Bp20Plus) are well described in the scientific literature, and are very distinctive from one another, and this was reflected in the plots of their 90% duration and 5th–95th percentile frequency bounds. In contrast, the properties of Bm-D and Bp-Downsweep, have not been as well defined in the literature and have forms that appear very similar to each other. Thus these classes have higher potential for confusion and a higher likelihood of being marked as unidentified. As a result, the time–frequency bounds of unidentified calls combined two categories: (1) calls that clearly did not fit into any of the defined classifications, and (2) calls that were intermediate between Bm-D and Bp-Downsweep. However, by restricting annotations to only signals that can be definitively attributed to one species or the other, they do appear to be distinguishable using duration and frequency (Fig. 5). This is an instance where having multiple experienced analysts annotate the same data set might converge on clear guidelines for distinguishing between the two call types. While decisions to only annotate or detect signals that are clearly attributable to a known species are necessary and justifiable, further research on acoustic behaviour would be required to determine whether this has downstream implications for making accurate population abundance estimates.

In contrast to the duration measurements, the upper frequency limit of the Bp-20Plus call type did show true differences across sites revealing geographic separation similar to that which has been described in previous studies^15,18^. Gedamke (2009)^15^ found that fin whales detected on recorders in the Indian Ocean (including sites south of 60°S had higher-frequency components near 100 Hz, while fin whales detected in the Tasman Sea (Pacific Ocean including sites south of 60° S) had higher frequency components at 82 and 94 Hz. Širović et al. (2009)^18^ found that fin whale sounds recorded off the WAP and Scotia Sea had higher frequency components around 90 Hz, while recordings off East Antarctica had higher frequency components near 100 Hz. In our study, the Indian and Atlantic sectors had higher frequency components around 100 Hz, while the WAP and Pacific sectors were around 90 Hz. Recordings investigated by Gedamke (2009)^15^ and Širović et al. (2009)^18^ were made from 2003 to 2007, whereas all but one of our recordings were made in 2013–2017. Thus, there appears to be decadal-scale stability in the broad geographic distribution and form of these sounds.

### Temporal distribution of annotations within a site-year

**Figure 6:**
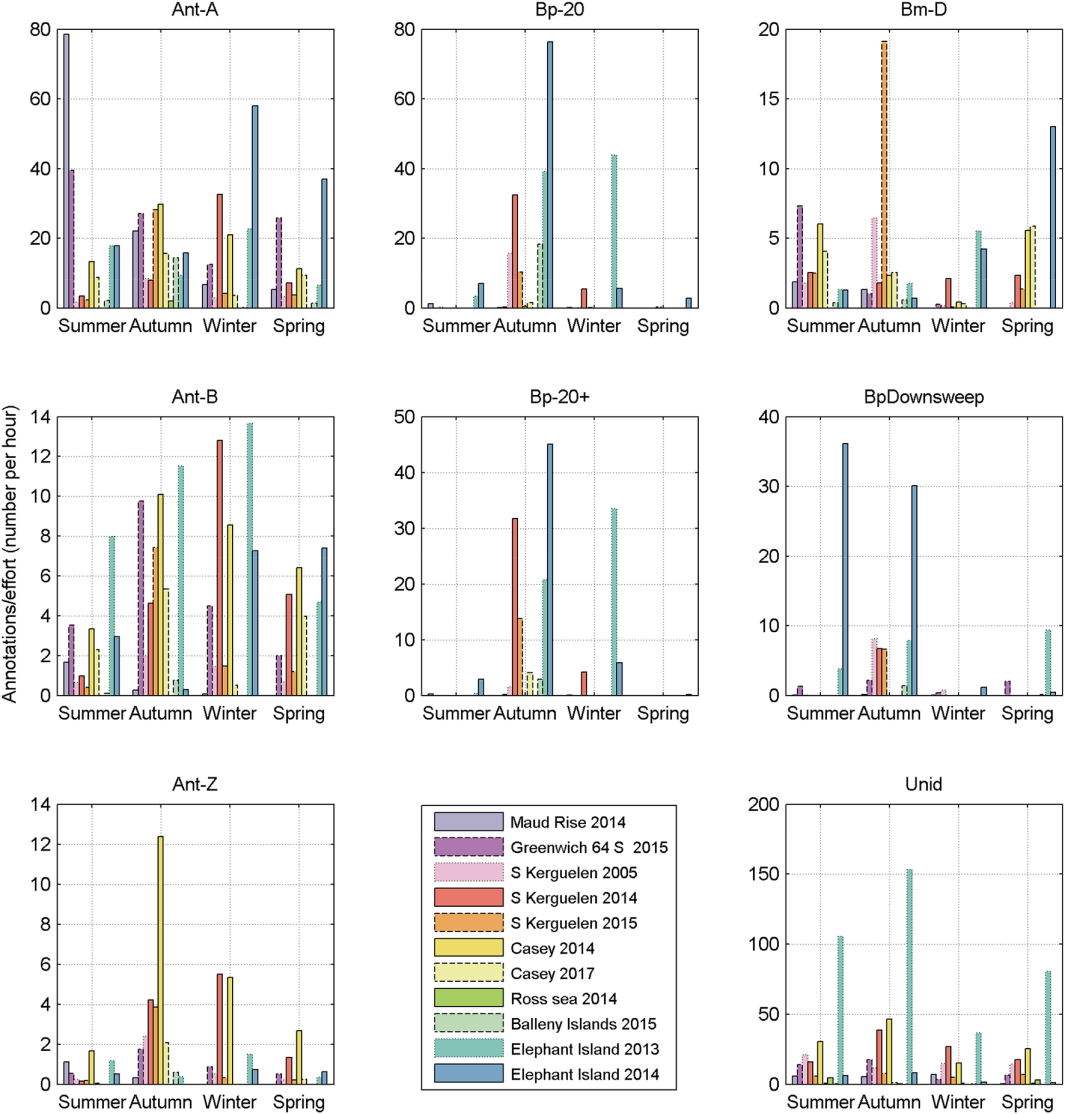
Rate of annotations per season for each site and sound type. Rate is calculated as the total number of annotations in that season divided by the total effort (in hours) for that season. Antarctic blue whale tonal-sounds are in the left column. Fin whale 20 Hz pulses are in the middle column. Blue D, fin downsweeps, and unidentified calls are in the third column. Vertical scale may differ for each panel.

In general there were more annotations from Feb through May (late summer through autumn) than in other months, though there were a number of exceptions to this general trend (Fig. 6). Bm-Ant-A had maximum number of annotations from June–August at Elephant Island 2014 and Kerguelen 2014. Bm-Ant-Z also peaked in July at Kerguelen 2014. At Casey 2014 Bm-D had a maximum in December, while at Elephant Island 2014 Bm-D had maximum monthly annotations in October. At Elephant Island 2014 Bp-Downsweep had a maximum in January.

While there is a temptation to speculate on the drivers of these temporal trends, such analyses are beyond the scope of this work, which was the creation of a dataset suitable for characterising automated detectors. Rather, the purpose of plotting monthly number of annotations by site is simply to describe the contents of the Annotated Library and to identify months or seasons that do and do not have sufficient number of detections to allow characterisation of a detector. In that regard, there is a notable lack of fin whale annotations (Bp-20 Hz, Bp-20Plus, and Bp-Downsweep) from July-December.

### An example of using the annotated data to examine the performance of automated detectors

#### ROC, precision-recall, and SNR

**Figure 7:**
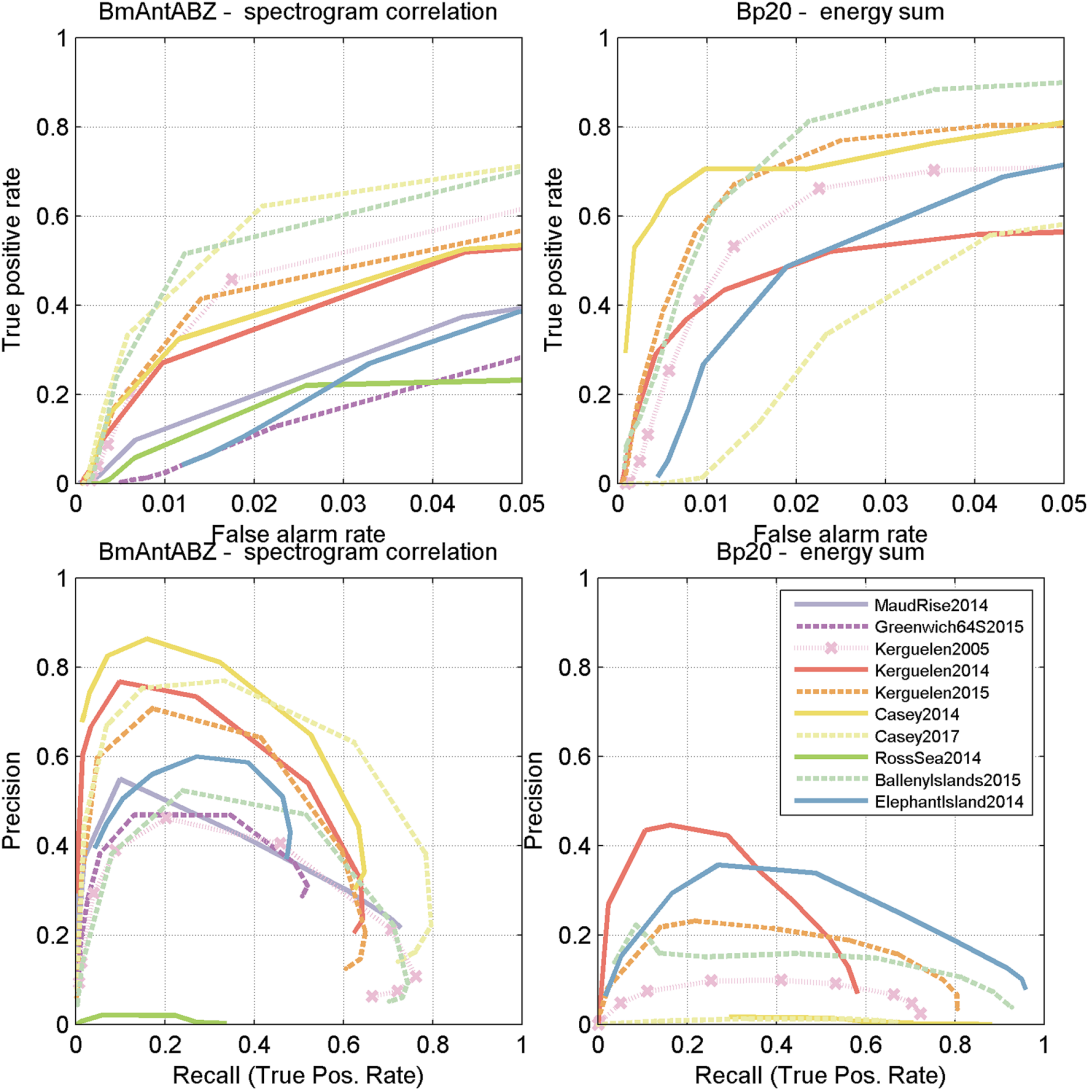
ROC curves (top), and precision-recall curves (bottom) for Bm-Ant-A; Bm-Ant-B; Bm-Ant-Z spectrogram correlation detector (left) and Bp-20 energy detector (right). Sites with fewer than 30 detections of Bp20 (Maud Rise 2014, Greenwich64S 2015, Casey 2014, Ross sea 2014) are not shown.

ROC and PR curves indicated that detector performance was fair-to-poor for these datasets. ROC and PR curves varied by site for both blue and fin whale detectors with some sites much worse than others (Fig. 7). For example, the true positive rate for the blue whale detector ranged from 8 to 55% at a false alarm rate of 1% (~ 2.8 false positives per hour). The true positive rate for the fin whale detector ranged from 1 to 76% at a false alarm rate of 1% (~ 14.4 false positives per hour).

**Figure 8:**
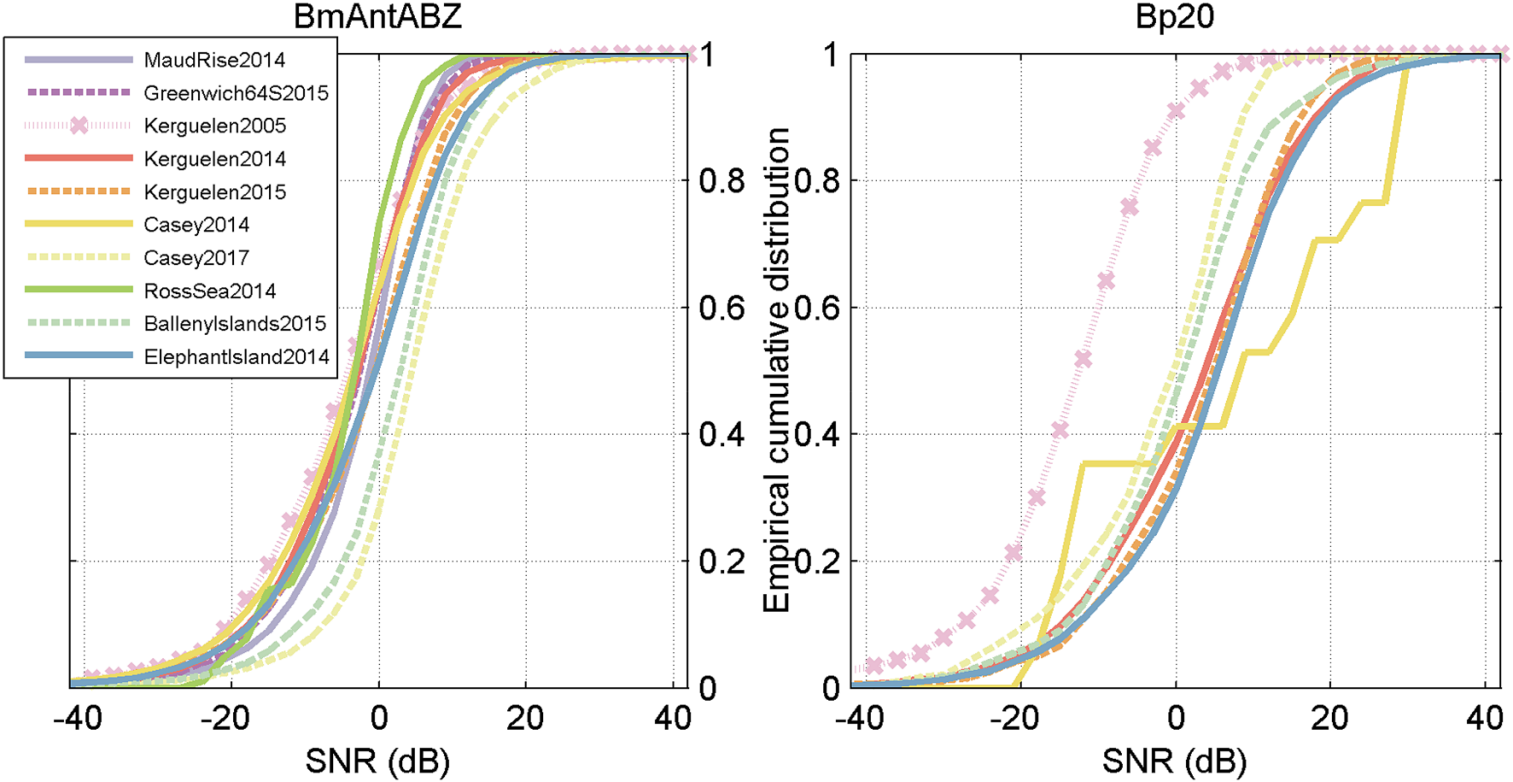
Empirical cumulative distribution of signal-to-noise ratio (SNR) of manually annotated blue whale song (left) and manually annotated fin whale pulses (right) for each site. Blue whale distributions include calls classified as Bm-Ant-A, Bm-Ant-B, or Bm-Ant-Z. Fin whale distributions includes calls classified as Bp-20 Hz or Bp-20Plus.

**Figure 9:**
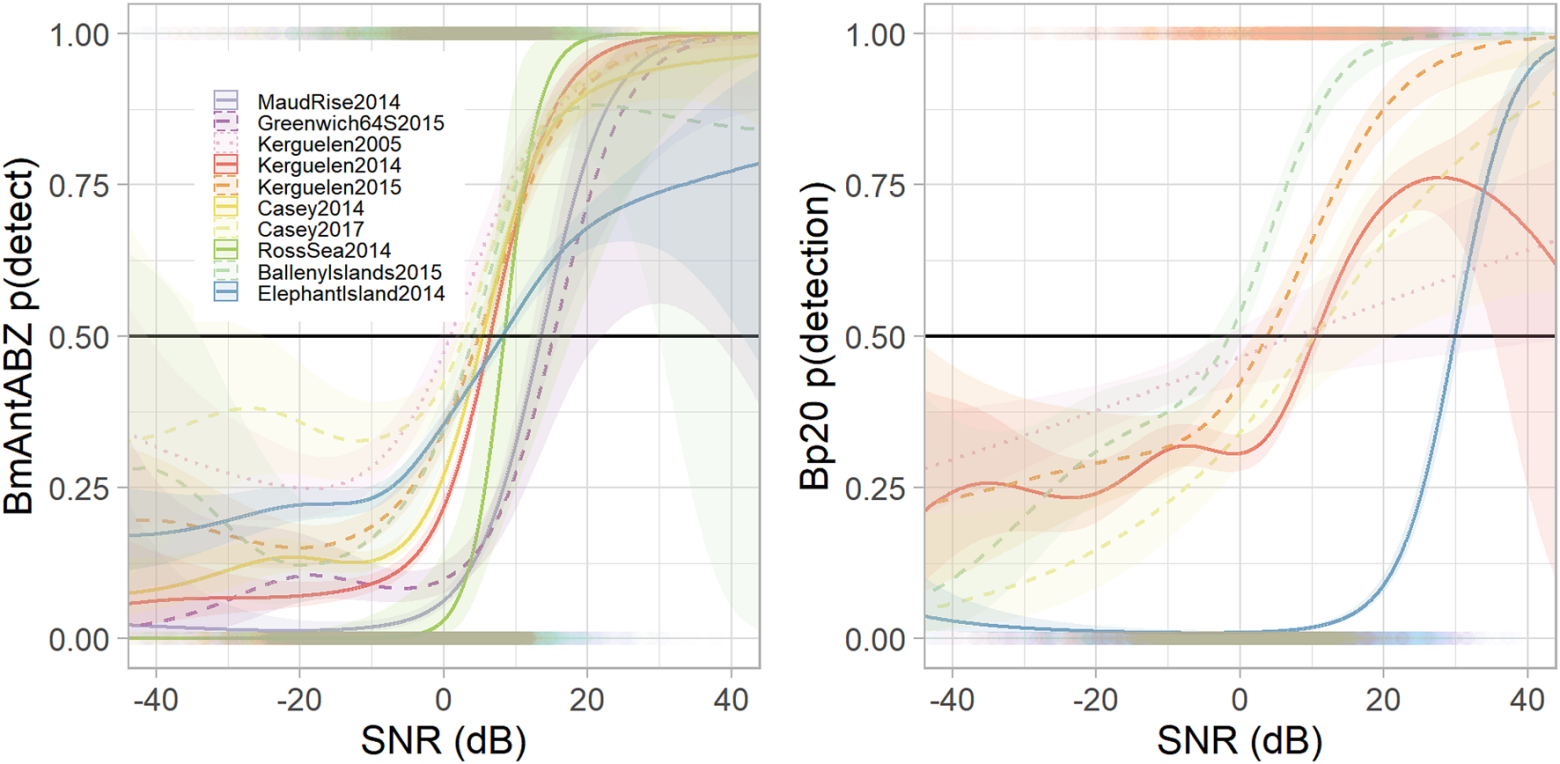
Probability of automated detection of annotated call as a function of SNR. Specifically, these are the marginal effects for SNR from the binomial GAM from Eq. (3). Left: blue whale annotations (any of Bm-Ant-A, Bm-Ant-B, Bm-Ant-Z) for the spectrogram correlation detector using the theshold nearest to false positive rate of 0.01. Right: Bp-20 annotations (either Bp-20 Hz or Bp-20Plus) for the energy sum detector with the threshold nearest to false positive rate of 0.01. Shading shows 95% confidence intervals for each site, and rug plots show the distribution of data as.

In addition to variability in detector performance, the distribution of SNR also varied across sites with the combined Bp20 and Bp20 plus distributions showing more variability than the combined Bm-Ant-A, Bm-Ant-B, and Bm-Ant-Z distributions (Fig. 8). The modelled probability of detection at 1% false positive rate was similar across sites at high-SNR, but was more variable across sites at low SNR (e.g. < 0 dB) (Fig. 9).

## Discussion

We created an annotated library of blue and fin whale sounds that spans four circumpolar Antarctic recording regions, five different years (2005, 2013, 2014, 2015, 2017), and five different types of instrument. The acoustic data in our library come from a variety of different data collection campaigns conducted by laboratories from five nations.

The distribution of calls in our library varied considerably across sites, years, and species. Antarctic blue whale sounds, particularly Bm-Ant-A, were the most numerous, and are well represented at all sites, and over most times throughout the year. Fin whale sounds had a much more seasonal representation in the annotated library with annotations in late summer and throughout autumn months, and few throughout the rest of the year. Fin whale Bp-20Plus sounds also revealed some degree of biogeographic separation with calls in the Atlantic and Indian sectors having higher upper-frequency components than those in the Pacific and WAP sectors. The annotations in the library form a representative ground-truth dataset that can be used to extract the features of each call type, and also to train and characterise the performance of automated detectors.

### Detector performance

To test the utility of the library, we characterised the performance of a spectrogram correlation detector for blue whale calls and an energy sum detector for fin whale calls. The performance of the automated detectors varied by site-year. Neither detector performed particularly well, and some sites and years showed much worse performance than others (Fig. 7). Differences in detector performance broadly followed differences in SNR across sites such that sites with lower SNR had worse performance than those with higher SNR (Fig. 8). Across sites, the automated detectors showed greater variability at low SNR than at high SNR (Fig. 9).

Characterising the performance of an automated detector and estimating the probability of automatic detection as a function of SNR using a representative subset of data, as we have done here, can be important steps towards meaningful comparisons of animal sounds across sites and over time^43,46^. In addition to performance of the detector, differences in call density (a useful metric for such comparisons) can arise from site-specific factors such as differences in instrumentation (including depth)^54^, analyst variability^53,65^, ambient and local noise sources^36,66^, propagation^46,67^, and animal behaviour^43^. These factors are not mutually exclusive, and can interact in a complex manner. Addressing and accounting for how each of these factors affects the call density is beyond the scope of this manuscript, but is a requirement if one wants to make comparisons of acoustic detections that meaningfully address biological questions of distribution and temporal trends. The library and methods we present here for assessing the performance of the detectors are a step away from estimating call-density, which in turn is a step away from estimating animal density^68^.

None of the passive acoustic studies of Antarctic blue or fin whales to date (listed in Table 1) have completely reported on the performance of their detector over a representative subsample of their data. The methods we have presented here for characterising the performance of a detector on a representative subsample of data constitute a bare minimum of reporting for future studies that utilise automated detectors to study Antarctic blue and fin whale calls. Specifically, reporting should include all parameters for the automated detector including any noise pre-processing steps; distribution and SNR of ground-truth detections throughout the dataset; and true and false positive rates and/or precision and recall of the detector for a representative sample of the data.

We hope the open-access annotated library we have presented here can provide a base dataset upon which to develop improved detectors i.e. with higher true positive rates and lower false positive rates. Here we have extracted duration and frequency measurements from annotations, but the library can readily be used to extract more complex features such as pitch-tracks^69^ or other time–frequency features^70^ to train machine learning algorithms^37,71,72^, deep neural networks^73^, or other any other advanced detectors that may provide better performance than the spectrogram correlation detector. Better detectors would not only reduce a source of uncertainty in estimating call-density, but would also reduce the amount of analyst effort required to verify true positives and account for false positives.

Future development of this dataset will aim to expand the annotated library to serve as a test-bed for subsequent analyses that address the issues of noise, detection range, and analyst variability to produce standardised outputs that are appropriate for circumpolar comparisons of call-density. This additional development would entail (1) collating pressure calibration details for noise analysis at each site-year, (2) estimating detection range throughout each site-year and (3) having multiple analysts annotate the same subsets of data for the purposes of quantifying analyst bias and variability.

## Conclusions

We created an annotated library of blue and fin whale sounds that spans four circumpolar Antarctic recording regions, five different years (2005, 2013, 2014, 2015, 2017), and five different types of instrument. The annotations in the library form a representative ground-truth dataset and we demonstrate how to train, test, and characterise the performance of two common automated detectors using the library. The annotated library we present here can serve as a benchmark upon which detectors can be developed, compared, and improved upon. It may also serve as a base dataset to develop additional analytical techniques to enable robust comparisons of acoustic detections of blue and fin whale across diverse circumpolar sites and over long spans of time.

We encourage further contributions of data and annotations to help expand the library, and in the future hope to include annotations of sounds from additional Antarctic species, as well as data from other recording locations throughout the southern hemisphere. The IWC-SORP/SOOS Acoustic Trends Annotated Library is freely available from http://data.aad.gov.au/metadata/records/AcousticTrends_BlueFinLibrary^74^. The larger datasets from which the Annotated Library was derived are available under the data sharing provisions of the Antarctic Treaty (1959), and these can be requested by contacting the authors and/or institutions that hold these data.

## Data Availability

Code available in the IWC-SORP/SOOS Annotated Library (https://data.aad.gov.au/metadata/records/fulldisplay/AcousticTrends_BlueFinLibrary).

## Data availability

Data used in this study are publicly available under a Creative Commons 4.0 Attribution licence. They can be accessed via the Australian Antarctic Data Centre at http://data.aad.gov.au/metadata/records/AcousticTrends_BlueFinLibrary.
